# Astrocytes require insulin-like growth factor I to protect neurons against oxidative injury

**DOI:** 10.12688/f1000research.3-28.v2

**Published:** 2014-04-22

**Authors:** Laura Genis, David Dávila, Silvia Fernandez, Andrea Pozo-Rodrigálvarez, Ricardo Martínez-Murillo, Ignacio Torres-Aleman

**Affiliations:** 1 Instituto Cajal CSIC, 28002, Madrid, Spain; 2 CIBERNED, 28002, Madrid, Spain

## Abstract

Oxidative stress is a proposed mechanism in brain aging, making the study of its regulatory processes an important aspect of current neurobiological research. In this regard, the role of the aging regulator insulin-like growth factor I (IGF-I) in brain responses to oxidative stress remains elusive as both beneficial and detrimental actions have been ascribed to this growth factor.

Because astrocytes protect neurons against oxidative injury, we explored whether IGF-I participates in astrocyte neuroprotection and found that blockade of the IGF-I receptor in astrocytes abrogated their rescuing effect on neurons. We found that IGF-I directly protects astrocytes against oxidative stress (H _2_O _2_). Indeed, in astrocytes but not in neurons, IGF-I decreases the pro-oxidant protein thioredoxin-interacting protein 1 and normalizes the levels of reactive oxygen species. Furthermore, IGF-I cooperates with trophic signals produced by astrocytes in response to H _2_O _2 _such as stem cell factor (SCF) to protect neurons against oxidative insult. After stroke, a condition associated with brain aging where oxidative injury affects peri-infarcted regions, a simultaneous increase in SCF and IGF-I expression was found in the cortex, suggesting that a similar cooperative response takes place *in vivo*. Cell-specific modulation by IGF-I of brain responses to oxidative stress may contribute in clarifying the role of IGF-I in brain aging.

## Introduction

Oxidative stress is usually considered a mechanism of brain aging ^1^. However, contradictory data ^2^ and lack of firm evidence ^3^ makes it difficult to firmly establish its actual significance in this process (see López-Otín *et al.* ^4^ for a recent review). One important aspect that requires further clarification in this regard is the relationship between oxidative stress and insulin peptides, a well conserved family of hormones firmly linked to aging. Extensive work in vertebrates and invertebrates indicates that the insulin-like growth factor I (IGF-I)/insulin signalling (IIS) pathway has a negative impact on aging. It has been argued that this detrimental action is mediated by reducing cell defences to oxidative stress ^5– 7^ which, in turn is harmful for neuronal survival ^1^. However, IGF-I has been shown to be largely neuroprotective ^8^, even in conditions such as ischemic injury or brain trauma where oxidative stress is most likely a major pathogenic mechanism ^9^. Thus, it is unclear whether or not IGF-I protects the brain against oxidative stress as the current evidence is contradictory.

A possible explanation for these apparently contradictory observations may be that modulation of the cellular response to oxidative stress by IGF-I is cell-dependent ^10^. Until now, only neurons have been studied in this regard. However, astrocytes, a major cellular element of the brain, are essential contributors to neuronal homeostasis and are coupled to neurons in the response to oxidative stress in order to help protect them ^11^. It is thus possible that IGF-I participates in the response of astrocytes to oxidative stress as part of the overall brain response encompassing all types of brain cells, not only neurons. Contrary to what we previously observed in neurons ^12^, we report here that IGF-I protects astrocytes against oxidative stress and, very significantly, also co-operates with astrocytes to protect neurons.

## Methods

### Animals

We used postnatal rats and mice for *in vitro* cultures (P0-3 days for astrocytes and P7 for neurons) and 3 month old mice for *in vivo* experiments. P2 Wistar rats (8 g ± 0.04 body weight, n=240, Harlan, Spain), P3 (2 g ± 0.03, n=36, Harlan) and 3 months old (27.6 g ± 0.812; n= 24) C57BL6 mice and P7 GFP transgenic mouse pups (4.25 g ± 0.22, n=126; in-house colony) were used. Pups used were of both sexes and no attempt to sex them was done. Adult mice were male. Rat tissue was used in all *in vitro* experiments except when using GFP cell derived from transgenic mice. *In vivo* experiments were done in mice for future comparisons with transgenic mice. All efforts were made to minimize suffering and reduce the number of animals. Animals were kept under light/dark, 12 h/12 h) conditions following EU guidelines (directive 86/609/EEC) and handled according to institutionally-approved procedures (CSIC bio-ethics subcommittee project code SAF2010-1703). Animals were fed ad libitum with laboratory rodent chow (Teklad Global 2018S) and kept in standard laboratory cage conditions with 4 animals/cage.

### Reagents

Antibodies used in this study are detailed in Table 1. The different drug inhibitors used in the study are given in Table 2. Hydrogen peroxide (H _2_O _2_) and the calcium chelator BAPTA-AM were purchased from Sigma (Steinheim, Germany). IGF-I and SCF were purchased from Prospec-Tany Technogene, (Israel).

**Table 1.** Antibodies used in the study.

ANTIBODY	PRODUCT Nº	MANUFACTURER	WORKING CC	SPECIES	ISOTYPE	ANTIGEN (EPITOPE)	AFFINITY PURIFIED	REFERENCE
Akt1/2 (H-136)	sc-8312	Santa Cruz Biotechnology (California, USA)	1:1000	rabbit	polyclonal	aminoacids 345–480 of human Akt1/2	unknown	^42– 45^
β-actin (Clone AC-74)	A5316	Sigma (Steinheim, Germany)	1:50000	mouse	monoclonal	N-terminal end of β-isoform of actin	ascytes fluid	^46– 49^
Cu/Zn superoxide dismutase (SOD)	SOD-101	Assay Designs (Michigan, USA)	1:1000	rabbit	polyclonal	Native rat Cu/Zn SOD	yes	^50, 51^
MnSOD superoxide dismutase (SOD)	SOD-111	Assay Designs (Michigan, USA)	1:2500	rabbit	polyclonal	Native rat Mn SOD	yes	^52– 55^
p44/p42 MAPK (ERK1/2)	9102	Cell Signalling (Danvers, USA)	1:2000	rabbit	polyclonal	sequence in the C-terminal of rat p44MAPK	yes	^56– 59^
phospho-Akt (Ser473)	9271	Cell Signalling (Danvers, USA)	1:1000	rabbit	polyclonal	residues surrounding Ser473 of mouse Akt	yes	^46, 60– 63^
phospho- ERK1/2 (Thr202/Tyr204)	9101	Cell Signalling (Danvers, USA)	1:2000	rabbit	polyclonal	residues surrounding Thr202/Tyr204 of human p44 MAPK	yes	^63, 63, 63– 66^
SCF	sc-9132	Santa Cruz Biotechnology (California, USA)	1:1000	rabbit	polyclonal	aminoacids 26–214 of human SCF	unknown	^67– 69^
TXNIP1	K0205-3	MBL (Nagoya, Japan)	1:2000	mouse	monoclonal	human recombinat TXNIP	unknown	no refs

**Table 2.** Drug inhibitors used in the study.

Target	Inhibitor	Dose	Supplier
CALCINEURIN	CYCLOSPORIN A	500 nM	Sigma-Aldrich
ERK MAPK	U0126	20 µM	Tocris Bioscience
Extracellular Ca2+	CdCl2/EGTA	100 µM/10 mM	Sigma-Aldrich
IGF-IR	PPP	120 nM	Calbiochem
Intracellular Ca _2+_	BAPTA/AM	5–10 µM	Calbiochem
JNK	Insolution ^TM^ JNK INHIBITOR II	10–20 µM	Calbiochem
mTOR	Insolution ^TM^ RAPAMYCIN	100 nM	Calbiochem
NF-KB	QNZ	10–20 nM	Enzo Life Sciences
p38 MAPK	SB203580 hydrochloride	20 µM	Calbiochem
PDK1	OSU-03012	10 µM	Echelon
PI3K	LY294002	25 µM	Calbiochem
PKA	KT5720	60 nM	Tocris Bioscience
PKC/PKA	Ro 31-8220	20–900 nM	Calbiochem
PKCα, PKCβI, PKCε	Rho 32-0432	0.2 µM	Calbiochem
PKC isotypes (α,β,γ,δ,ζ,µ)	Go6983	6 nM–20 µM	Tocris Bioscience
PP1	TAUTOMYCIN	2 nM	Calbiochem
PP2A	OKADAIC ACID (495609 Insolution)	2.5 nM	Calbiochem
PROTEASOME	MG-132	5 nM–3 µM	Calbiochem
PROTEIN SYNTHESIS	CYCLOHEXIMIDE	1 µg/ml	Calbiochem

### Plasmids

pECE-FOXO3 and pECE-FOXO3-TM (triple mutant T32A/S253A/S315A, herein called MFOXO3) were kindly provided by ME Greenberg (Harvard Medical School, Boston, USA). p6xDBE-luc (reporter luciferase plasmid with six copies of the DAF16 family protein-binding element) and pRL-TK (TK-Renilla luciferase) were a kind gift of BM Burgering (University Medical Centre, Utrecht, The Netherlands). Dominant negative IGF-IR expression plasmid was kindly donated by D. Le Roith (Mt Sinai, New York, USA). Plasmids expressing shRNA for TXNIP1 were purchased from Origene (USA). Txnip1 plasmid was purchased from Thermo Scientific Open Biosystems (Waltham, USA).

### Cell culture and transfections

Cerebellar granule cultures were produced from either P7 rat or GFP transgenic mouse cerebella as previously described ^13^. In brief, cells were plated onto 6 or 12-well dishes coated with poly- l-lysine (1 μg/ml) at a respective final density of 1.5×10 ^6^/well or 0.45×10 ^6^/well. Cells were incubated at 37°C/5% CO _2_ in Neurobasal (Gibco, USA) medium supplemented with 10% B27 (Gibco), glutamine (5 mM) and KCl (25 mM). All experiments were carried out in 2–7 day old cultures, with neurons showing neurite extensions. Different times *in vitro* were used to analyze time-dependent parameters such as cell survival. Rat granule neurons were transfected 24 h after plating. The DNA: transfection agent ratio (Neurofect, Genlantis, San Diego, USA) was 1:7. The percentage of neurons transfected was 5–10%, as assessed with a GFP vector. Neurons were left untreated for at least 48 hours. On the day of the experiment, medium was replaced with Neurobasal + 25 mM KCl. Two hours later, IGF-I (10 ^-7^ M) and/or hydrogen peroxide (H _2_O _2_) at doses of 50–150 µM were added. Inhibitory drugs were given 45 min before treatments. We used H _2_O _2_ as an oxidant stimulus because it is an endogenously produced reactive oxygen species (ROS) that serves as a precursor to hydroxyl radicals and possesses signalling capacities ^14^. Astrocyte cultures were prepared from P3 rat or GFP mouse forebrain, as previously described ^15^ after animals were sacrificed by decapitation. Cells were grown on Dulbecco’s modified Eagle's medium F12 (DMEM-F12) supplemented with 10% fetal calf serum. After 12 days astrocytes were seeded at 2.5×10 ^5^ or 1.25×10 ^5^ cells/well in 6-well and 12-well culture plates, respectively. On the day of the experiment cells were treated with IGF-I (10 ^-7^M), H _2_O _2_ (50–200 µM) and/or inhibitors, as above. For transfection, astrocytes were seeded at 2.5×10 ^5^ or 1.25×10 ^5^ cells/well in 6-well and 12-well culture plates respectively, and after 16 h constructs were mixed with Fugene HD (Roche, Switzerland) in a 1:3 ratio, and added following the manufacturer’s instructions. Alternatively, astrocytes were electroporated (2×10 ^6^ astrocytes with 2 µg DNA or shRNA) before seeding using an astrocyte Nucleofector Kit (Lonza, Switzerland). After electroporation, cells were plated to obtain a final cell density on the day of the experiment similar to that obtained with the transfection method. All experiments were performed after 48 h. The transfection efficiency was 20–30% and 60–80% for electroporation, as assessed with a GFP vector. At least three independent experiments were done in duplicate wells.

### Co-cultures

For co-cultures, 1.25×10 ^5^ wild type mouse astrocytes/well were seeded on 12-well plates and grown with DMEM-F12 plus 10% FBS. After 48–72 hours, GFP neurons were isolated and plated onto astrocytes. We used forebrain astrocytes and cerebellar neurons because in our experience the forebrain and cerebellum yielded very high numbers of astrocytes and neurons, respectively (thus minimizing animal use). Furthermore, in this study we were interested in exploring general, rather than region-specific neuroprotective characteristics of astrocytes. Nevertheless, we also carried out co-cultures with neurons and astrocytes from the same region (forebrain) and the results obtained were identical than when using cells from differing regions (see Figure 2 in results). Culture medium was changed to DMEM-F12 plus B27, 4 mM glutamine and 25 mM KCl (the latter only in the case of neurons). Two days later, co-cultures were treated with 100 nM IGF-I ± 50–100 µM H _2_O _2_. Pictures were taken every 24 hours up to 5–7 days as above. For protein silencing or overexpression, 2×10 ^6^ astrocytes were electroporated in a Nucleofector ^®^II (Amaxa Biosystems Lonza, Switzerland) and seeded at 1.25×10 ^5^/well. Co-cultured neurons were seeded as described above. Viability of neurons was assessed by counting the number of cells expressing GFP using Incucyte software (2010A) with a set cell size threshold to avoid including GFP ^+^ cell debris and dying cells. This threshold ranged from 8–36 µm ^2^ to 70–200 µm ^2^ depending on the experiment. Viability is expressed as percentage of GFP ^+^ cells at the beginning of the experiment (time 0). At least three independent experiments were done.

### Cell assays

Cell viability was determined by four different methods. The first assessed astrocyte death by quantification of the amount of lactate dehydrogenase (LDH) released from damaged astrocytes into the culture medium. LDH levels were measured after 16 h of treatment with different H _2_O _2_ concentrations using a commercial kit (Roche Diagnostics, Germany). When using transfected astrocytes, a GFP-pCMV vector and the different constructs under evaluation were used in a 1:5 ratio. In this case, GFP ^+^ astrocytes were scored prior to treatment to determine baseline survival (time 0) and at different times as indicated in the results. Alternative viability assays for astrocytes included measuring cell metabolism with fluorescein diacetate (0.1 µg/ml FDA) or number of propidium iodide (PI) cells ^12^ as specified in the results section. For the latter, astrocytes or neurons were stained with 2 µg/ml PI as a marker of dead cells plus DAPI staining as a marker of total cell number. PI ^+^ and DAPI ^+^ cells were counted under a Leica CTR 6000 fluorescence microscope. Percentage of viable cells indicates the number of PI ^+^ cells related to total cell number. The experiments were done in triplicate and a total of three independent experiments were done. For neuronal-specific viability assays cerebellar neurons from GFP mice were seeded on 12-well plates (4.5×10 ^5^ cells) coated with poly-L-Lysine and grown with Neurobasal medium plus B27, 4 mM glutamine and 25 mM KCl. After 4–5 days, cultures were treated with 100 nM IGF-I in the presence or absence of 50–100 µM H _2_O _2_. Pictures of GFP ^+^ cells (green fluorescence) were taken every 24 hours up to 3 days in an Incucyte ^TM^ 2010A Rev2 system (Essen BioScience, USA). Viability of neurons in co-culture experiments was measured as described above.

### Immunoassays

Western blotting was performed as described ^13^. Cells were washed once with ice-cold PBS and lysed with 1% NP-40, 150 mM NaCl, 20 mM Tris, pH 7.4, 10% glycerol, 1 mM CaCl _2_, 1 mM MgCl _2_, 400 μM sodium vanadate, 0.2 mM PMSF, 1 µg/ml leupeptin, 1 µg/ml aprotinin and 0.1% phosphatase inhibitor cocktails I and II (Sigma-Aldrich). To normalize for protein load, membranes were reblotted (Re-Blot, Chemicon, USA) and incubated with an appropriate control antibody (see Results). Levels of the protein under study were expressed relative to protein load. Different exposures of each blot were collected to ensure linearity and to match control levels for quantification.

Densitometric analysis was performed using Analysis Image Program (Bio-Rad, USA). A representative blot is shown from a total of at least three independent experiments. IGF-I levels in culture medium were measured using Quantikine ELISA for mouse/rat IGF-1 (R&D Systems, USA). In brief, cells were treated as described above and 1 ml of culture medium was collected after 24 hours, spun to eliminate cell debris, and stored at -80ºC. Samples were lyophilized overnight and resuspended in 150 µl of calibrator buffer. After vortexing, samples were centrifuged 10 min/14,000 rpm (Hettich, Germany) and assayed according to manufacturer’s instructions. A total of three and four independent experiments were done for neurons and astrocytes, respectively. SCF levels in culture medium were measured by western blot after collecting the supernatants and processing them as described above for IGF-I. After lyophilisation, samples were resuspended in western blot lysis buffer and protein levels were measured by Bradford (Biorad, Germany) following the manufacturer’s instructions to normalize for protein load in SDS-PAGE gels.

### Luciferase assays

Luciferase assays were done as previously reported ^12^. In brief, cells were transfected with a reporter construct bearing six canonical FOXO binding sites (6×DBE- luciferase) and co-transfected with different constructs, as indicated in each experiment. Transfections were performed in triplicate dishes. Luciferase counts were normalized using TK-Renilla luciferase. At given times, neurons were lysed in passive lysis buffer (PLB) and luciferase activity was analysed using a luminometer and dual luciferase assay kit according to the manufacturer (Promega, USA). Background luminescence was subtracted. Luciferase activity was expressed as fold of increase over control levels. At least three independent experiments were done.

### Flow cytometry

After 18h of exposure to H _2_O _2_, cell death was assessed. Cells were detached using 0.25% Trypsin-1.3 mM EDTA (Invitrogen) during 5–10 minutes, centrifuged (200× *g*, 5 min/4ºC), and resuspended in cold PBS. Propidium iodide (PI 5 µg/ml; Sigma) in PBS was added prior to flow cytometry analysis using a FACSAria cytometer (BD Biosciences). Fluorescence intensity, forward scatter (FSC), and side scatter (SSC) were collected in logarithmic scale. The emission filter used was 600–620 nm band pass (FL3). A fluorescence blank was measured and subtracted from the fluorescence of the sample. Dead cells were identified as red fluorescence positive events with low FSC (small PI permeable cells). Debris was always excluded from the analysis. At least three independent experiments were conducted.

### ROS measurement

Mitochondrial O _2_- production levels were measured by using the fluorescent probe MitoSOX ^TM^ Red (Life Technologies, USA). Briefly, astrocytes were pre-treated overnight with IGF-I and then 200 µM H _2_O _2_ were added during 1 hour. Cells were incubated with 1.5 µM MitoSOX ^TM^ Red in DMEM-F12 for 10 min/37ºC and washed 3 times with PBS. Astrocytes were then trypsinized and fluorescence was measured by flow cytometry (510 nm excitation/580 nm emission) using the cytometer, as described ^15^. A total of six independent experiments were done. Alternatively, ROS generation was assessed in astrocytes cultured on coverslips with the fluorogenic marker carboxy-H _2_DCFDA (Molecular Probes, USA) during 30 min/37ºC, protected from the light. When using this ROS marker it is not possible to distinguish endogenous ROS from exogenously applied H _2_O _2_. Nevertheless, we compared this method to the oxidation of luminol (which detects superoxide anions) that distinguishes H _2_O _2_ from other ROS and we obtained identical results with either method (data not visualized). The reason we used carboxy-H _2_DCFDA is because we could obtain both qualitative (cell images) and quantitative (fluorimetry assay) measurements within the same assay. After incubation with carboxy-H _2_DCFDA, cells were gently washed 3 times with warm DMEN, and mounted, or, alternatively, lysed for fluorimetry. Pictures were taken at 40× magnification using a Leica fluorescence microscope (Germany). A representative picture is shown. Fluorescence intensity in lysed cells was measured using a FluoroStar fluorimeter.

### Growth factor gene array

An RT ^2^ Profiler ^TM^ PCR Array (SABiosciences, USA) was used to screen a battery of growth factors following the manufacturer’s recommendations. After treatment, astrocytes were lysed and RNA extracted using Trizol (Life Technologies, USA). The resulting cDNA synthesis reaction was diluted in water, mixed with the qPCR master mix, and loaded in a 96 well PCR Array plate. PCR was performed following manufacturer’s instructions.

### Brain focal ischemia

Three-month old male mice (4–6 per group) were anesthetized with 3% isoflurane (in 70% N _2_O, 30% O _2_) for induction and with 2% isoflurane for maintenance. Rectal temperature was maintained at 36.5 C with a heating pad. The frontal branch of the medial cerebral artery (MCA) was exposed and occluded permanently by suture ligation as previously reported, with modifications ^16^. Briefly, an incision perpendicular to the line connecting the lateral canthus of the left eye and the external auditory canal was made to expose and retract the temporalis muscle. A burr hole was drilled, and frontal and parietal branches of the MCA were exposed by cutting and retracting the dura. The frontal branch of the MCA was elevated and ligated with a suture nylon monofilament 8/0. Following ligation, a sharp decrease of blood flow was evidenced with a laser Doppler flowmetry (Järfalla, Sweden). Following surgery, mice were returned to their cages, kept at room temperature and allowed free access to food and water. All physiological parameters measured: rectal temperature, mean arterial pressure and blood glucose levels were not different between groups. Sixteen hours after medial cerebral artery occlusion (MCAO), animals were killed by neck dislocation by an experienced researcher to assess infarct outcome. The brain was removed and the infarcted area isolated and processed for RNA and protein isolation.

### Quantitative PCR

Total RNA isolation from cell lysates or brain tissue was carried out with Trizol. One µg of RNA was reverse transcribed using High Capacity cDNA Reverse Transcription Kit (Life Technologies) according to the manufacturer’s instructions. For the quantification of specific genes, total RNA was isolated and transcribed as above and 62.5 ng of cDNA was amplified using TaqMan probes for Txnip1, IGF-I or SCF and 18S as endogenous control (Life Technologies). Each sample was run in triplicate in 20 μl of reaction volume using TaqMan Universal PCR Master Mix according to the manufacturer’s instructions (Life Technologies). All reactions were performed in a 7500 Real Time PCR system (Life Technologies). Quantitative real time PCR analysis was carried out as previously described ^17^. Results were expressed as relative expression ratios on the basis of group means for target transcripts versus reference 18S transcript. At least three independent experiments were done.

### Statistical analysis

Data are expressed as mean ± SEM. Differences among groups were analyzed by one- or two-way ANOVA followed by a Newman-Keul’s or Student’s t-test using Graph Pad Prism 5 software. A p<0.05 was considered significant.

## Results

### Astrocyte neuroprotection against oxidative stress requires IGF-I signalling onto astrocytes

**Figure 1.**
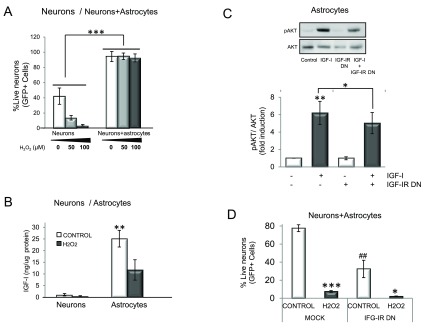
IGF-I signalling participates in astrocyte neuroprotection against oxidative injury. **A**) Neurons are protected from oxidative stress in the presence of astrocytes whereas when cultured alone they rapidly die. Viability of GFP neurons was measured as the number of green (GFP ^+^) cells two days after H _2_O _2_ treatment in the presence or absence of wild type astrocytes (F=41.85; ***p<0.001 vs. neurons alone. **B**) Both astrocytes and neurons secrete IGF-I, although astrocytes produce much higher levels (*p<0.05 vs neurons). H _2_O _2_ lowers IGF-I secretion. **C**) In the presence of a dominant negative IGF-IR (IGF-IR DN) signalling by IGF-I was markedly reduced. Astrocytes were transfected with IGF-IR DN or mock transfected, and the ratio pAkt/Akt (histograms) was measured as an index of IGF-I signalling. Representative blots and quantitative histograms are shown (2 way ANOVA, IGF-I and IGF-IR DN interaction: p<0.05, F=6.99; IGF-I p<0.01, F=13.46; IGF-IR DN p<0.05, F=7.06; Post-hoc: **p<0.01 vs control (mock-transfected) and *p<0.05 vs. IGF-I+IGF-IR DN). **D**) Blockade of IGF-IR function with IGF-IR DN compromises neuroprotection by astrocytes. GFP neurons were seeded on top of wild type astrocytes transfected with an IGF-IR DN construct or mock-transfected (control) and exposed to 100 µM H _2_O _2_. Viability of GFP neurons was measured after 5 days (2 way ANOVA, H _2_O _2_ and IGF-IR interaction: p<0.05, F=10.77; H _2_O _2_ p<0.01, F=68.92; IGF-IR DN p<0.05 F=17.86; post-hoc: ***p<0.001, *p<0.05 vs. Control; ##p<0.01 vs mock). Experiments were done at least 3 times in this and following figures. Bars are SEM in all figures.

Whereas neurons cultured without astrocytes are very sensitive to acute oxidative insult elicited by H _2_O _2_ ( Figure 1A), when cultured with astrocytes, neurons become very resilient ( Figure 1A). To determine whether IGF-I participates in the neuroprotective effects of astrocytes against oxidative stress we first confirmed that it is endogenously produced by these cells. As shown in Figure 1B, not only astrocytes but also neurons (albeit at much lower levels) secrete IGF-I into the culture medium. In response to H _2_O _2_ astrocytes secrete lower, but still substantial, amounts of IGF-I, and so IGF-I may still participate in neuroprotection by astrocytes. To directly test this possibility we blocked IGF-I signalling in astrocytes with a dominant negative (DN) IGF-IR ^18^ ( Figure 1C) and determined their ability to protect neurons against oxidative challenge. As shown in Figure 1D, a significantly greater percentage of neurons co-cultured with mock-transfected astrocytes survived after H _2_O _2_ challenge than when cultured with astrocytes transfected with DN IGF-IR.

**Figure 2.**
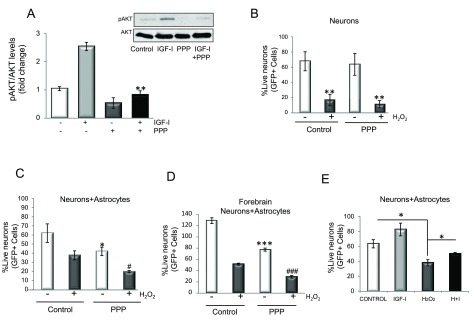
Endogenously produced IGF-I protects neurons against oxidative injury. **A**) The IGF-IR inhibitor PPP blocks IGF-I signalling in astrocytes. Astrocytes were treated with 120 nM PPP 1h before adding IGF-I while pAkt levels were measured 10 minutes after adding IGF-I. Ratios are shown in histograms (2 way ANOVA, IGF-I and PPP interaction: p<0.01, F=33.07; IGF-I p<0.01, F=27.38; PPP p<0.001, F=112.3; post-hoc: **p<0.01 vs. IGF-I alone). Representative blot is shown. **B**) Blockade of IGF-IR signalling with PPP in neurons cultured alone does not affect H _2_O _2_ toxicity after 3–4 days of exposure (2 way ANOVA, H _2_O _2_ and PPP interaction: F=0.069; H _2_O _2_ p<0.01, F=12.43; PPP F=3.66; post-hoc: **p<0.01 vs. respective controls). Note that PPP alone does not affect neuronal survival. **C**) Viability of cerebellar neurons co-cultured with forebrain astrocytes decreased significantly when treated with PPP for six days. PPP treatment in the presence of H _2_O _2_ decreased neuronal viability even further (2 way ANOVA, H _2_O _2_ and PPP interaction: F=0.097; H _2_O _2_ p<0.05, F=9.65; PPP p<0.01, F=31.33; post-hoc: *p<0.05 vs untreated control and #p<0.05 vs H _2_O _2_). **D**) Viability of forebrain neurons co-cultured with forebrain astrocytes decreased significantly when treated with PPP for five days. PPP treatment in the presence of H _2_O _2_ decreased neuronal viability even further (2 way ANOVA, H _2_O _2_ and PPP interaction: p<0.001, F=23.74; H _2_O _2_ p<0.001, F=321.6; PPP p<0.001, F=151.3, post-hoc: ***p<0.01 vs. untreated control and ### p<0.01 vs. H _2_O _2_). **E**) When co-cultured with wild type astrocytes, neuronal survival after five days of exposure to 100 µM H _2_O _2_ was moderately increased in the presence of 100 nM IGF-I (2 way ANOVA, H _2_O _2_ and IGF-I interaction: F=0.542; IGF-I p<0.05, F=7.28; H _2_O _2_ p<0.001, F=25.9; post-hoc: *p<0.05 vs. control or H _2_O _2_). I+H: IGF-I + H _2_O _2_.

We next used pharmacological blockade of the IGF-I receptor using picropodophyllin (PPP), an antagonist of IGF-IR ( Figure 2A). As in this case both the neuronal and astrocyte receptors are blocked, we first determined whether neurons are affected by PPP blockade of the IGF-I receptor. In the presence of H _2_O _2_, neurons cultured alone die regardless of the presence or absence of proper IGF-I signalling since PPP did not increase neuronal death ( Figure 2B). This agrees with our previous findings that IGF-I does not protect cultured neurons against oxidative stress ^12^. Confirming the results seen with astrocytes transfected with dominant negative IGF-I receptor, a reduction in neuroprotection by astrocytes was seen when co-cultured neurons were exposed to PPP. In the presence of H _2_O _2_, significantly fewer co-cultured neurons survived with PPP (p<0.01 H _2_O _2_+PPP vs. H _2_O _2_ alone; Figure 2C). To rule out region-specific actions of astrocytes on neuroprotection we then co-cultured neurons and astrocytes from the same brain region (forebrain) and treated them with PPP. As shown in Figure 2D, forebrain neurons were similarly sensitive to blockade of IGF-IR when co-cultured with forebrain astrocytes. The observation that even supra-physiological doses of IGF-I (100 nM) added to the co-cultures only produced a modest additional effect on neuronal survival after oxidative insult confirmed the idea that endogenous IGF-I is required by astrocytes for neuroprotection ( Figure 2E). Hence, endogenous production of IGF-I is necessary and sufficient to protect neurons.

### IGF-I protects astrocytes against oxidative stress

**Figure 3.**
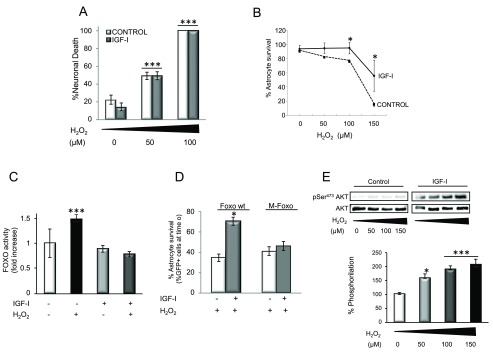
IGF-I protects astrocytes against oxidative stress. **A**) Whereas IGF-I increases neuronal survival under control conditions, it does not protect neurons from H _2_O _2_ induced death. This confirms previous observations ^12^. Neuronal mortality was measured by counting PI ^+^ cells 6h after treatment. H _2_O _2_ induces neuronal death in a dose-dependent manner irrespective of the presence of IGF-I (2 way ANOVA, H _2_O _2_ and IGF-I interaction: p<0.001, F=10.3; IGF-I p<0.05, F=9.98; H _2_O _2_ p<0.001, F=128.7; post-hoc: ***p<0.001 vs. no H _2_O _2¸_, # p<0.05 vs control). **B**) IGF-I treatment protects astrocytes from H _2_O _2_ induced death. Astrocyte demise was measured by counting PI ^+^ cells 24 h after H _2_O _2_ (100 µM). H _2_O _2_ exerts a dose-dependent effect that is reduced by IGF-I (2 way ANOVA, H _2_O _2_ and IGF-I interaction: p<0.01, F=5.36; IGF-I p<0.001, F=30.29; H _2_O _2_ p<0.001, F=60.42; post-hoc: *p<0.05 vs control). **C**) IGF-I blocks FOXO activity induced by H _2_O _2_ (100 µM). FOXO activity was measured with a luciferase reporter in astrocytes treated with IGF-I, H _2_O _2_ or both for 24 h (2 way ANOVA, H _2_O _2_ and IGF-I interaction: p<0.001, F=25.98; IGF-I p<0.001, F=49.58; H _2_O _2_ p<0.01, F=10.47; post-hoc: ***p<0.001 vs no treatment). **D**) Protection by IGF-I against cell death induced by H _2_O _2_ requires blockade of FOXO activity. Astrocyte viability was measured by counting GFP ^+^ astrocytes after co-transfection of GFP and a FOXO wild type (wt) or an Akt-insensitive mutant of FOXO (M-FOXO; 2 way ANOVA, M-FOXO and IGF-I interaction: p<0.01, F=59.99; IGF-I p<0.05, F=13.31; M-FOXO p<0.01, F=21.84; post-hoc: *p<0.05 vs no IGF-I). **E**) IGF-I increases phosphorylation of Akt (pAkt) in the presence of H _2_O _2_ in a dose-dependent fashion. Representative blots are shown. Lower histograms indicate quantification of pAkt/Akt ratio in the presence of IGF-I as shown in the right blot. pAkt levels were measured after 15 min. (*p<0.05 and ***p<0.001 vs. no H _2_O _2_).

**Figure 4.**
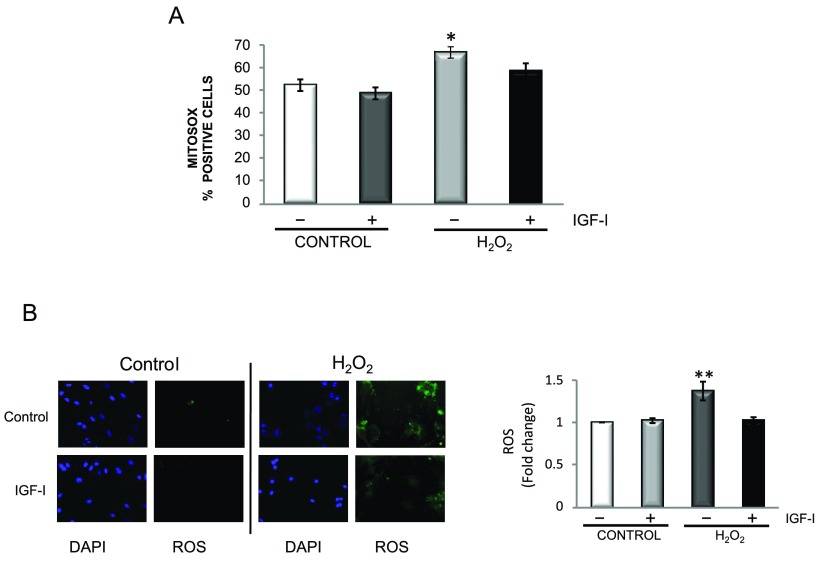
IGF-I reduces oxidative stress in astrocytes. **A**) H _2_O _2_ increases the number of astrocytes expressing mitochondrial O _2_^-^. This increase is prevented when cells are pre-treated with IGF-I. Mitochondrial O _2_^-^ levels were detected with MitoSOX by flow cytometry. Astrocytes were treated overnight with IGF-I and for 1 hour more with 200 µM H _2_O _2_ (2 way ANOVA, H _2_O _2_ and IGF-I interaction: F=1.27; IGF-I p<0.05, F=8.18; H _2_O _2_ p<0.01, F=16.18; post-hoc: **p<0.01 H _2_O _2_ vs control, *p<0.05 H _2_O _2_ vs IGF-I + H _2_O _2_). **B**) IGF-I lowers ROS levels after treatment of astrocytes with H _2_O _2_ (100 µM). Left: representative photomicrographs of astrocytes stained with carboxy-H _2_DCFDA to detect ROS and DAPI to stain cell nuclei. The increase in fluorescent cells elicited by H _2_O _2_ was markedly diminished by IGF-I. Right histograms: fluorimetric quantification of ROS levels with carboxy-H _2_DCFDA confirmed the rescuing action of IGF-I on astrocytes exposed to H _2_O _2_. (2 way ANOVA, H _2_O _2_ and IGF-I interaction: p<0.05, F=7.38; IGF-I p<0.05, F=5.89; H _2_O _2_ p<0.05, F=8.49; post-hoc: **p<0.01 H _2_O _2_ vs control, IGF-I, or IGF-I + H _2_O _2_).

IGF-I-dependent neuroprotection by astrocytes appears to also involve a direct action of IGF-I on astrocytes. Because it is known that astrocytes are more resistant to oxidative damage than neurons, we explored whether IGF-I was involved in this greater resilience. Contrary to neurons ( Figure 3A), IGF-I protected astrocytes against H _2_O _2_-induced death ( Figure 3B). The protective effect of IGF-I involved blockade of the activation of FOXO 3, a transcription factor involved in brain responses to oxidative stress ^19^, by H _2_O _2_ ( Figure 3C). Inhibition of FOXO 3 by IGF-I was mediated by Akt; i.e.: an Akt-insensitive mutant of FOXO (M-FOXO3) abrogated IGF-I effects while wild type FOXO3 did not interfere with its protective actions ( Figure 3D). Indeed, in astrocytes IGF-I activates Akt in the presence of H _2_O _2_ (Figure 3E), whereas in neurons H _2_O _2_ blocks this canonical pathway ^12^. Underlying the protective actions of IGF-I on astrocytes was its ability to block excess ROS after exposure to H _2_O _2_ as determined by flow cytometry using MitoSOX ( Figure 4A) or fluorometry with carboxy-H _2_DCFDA ( Figure 4B).

**Figure 5.**
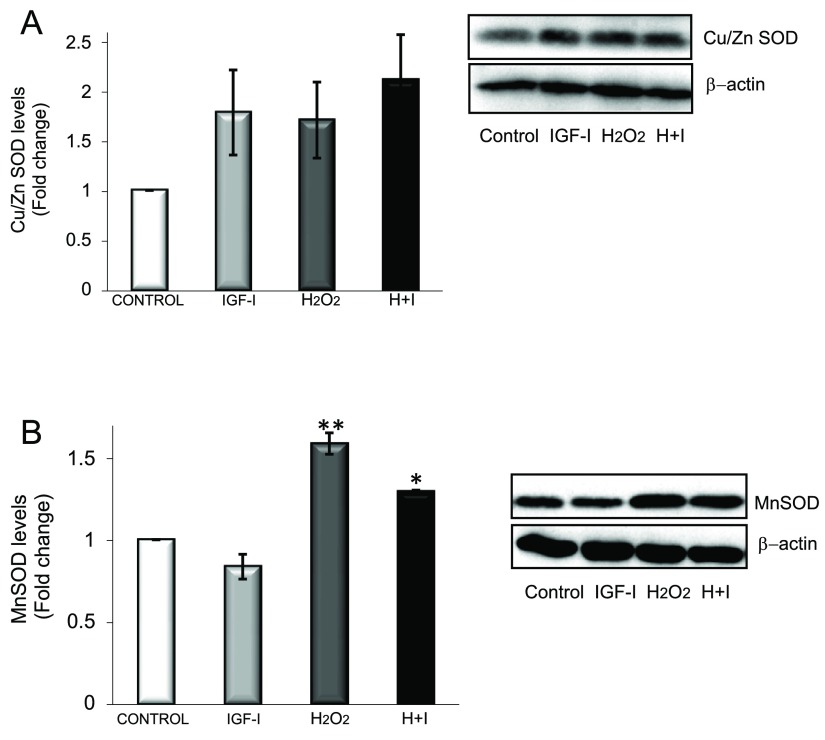
SOD responses to oxidative stress in astrocytes. **A**) Cu/ZnSOD levels in astrocytes are modulated by IGF-I and H _2_O _2_. **B**) MnSOD levels are enhanced by H _2_O _2_ but not by IGF-I (*p<0.05 and **p<0.01 vs control).

**Figure 6.**
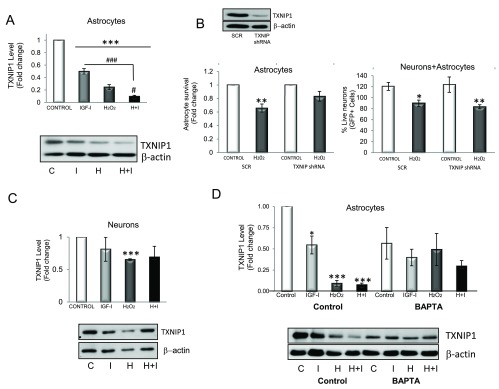
Both H _2_O _2_ and IGF-I reduce TXNIP1 in astrocytes. **A**) Levels of the pro-oxidant protein TXNIP1 are reduced by IGF-I and H _2_O _2_. Inhibition is greater when both are added together (F=156.6; ***p<0.001 vs. control and ###p<0.001 (vs. IGF-I) and #p<0.05 (vs. H _2_O _2_). Levels of actin in each sample were measured to normalize TXNIP1 levels. **B**) Western blot: transfection of astrocytes with shRNA TXNIP1 results in reduced TXNIP1 levels as compared to astrocytes transfected with scrambled shRNA (SCR). Left panel: TXNIP1 shRNA silencing makes astrocytes less sensitive to H _2_O _2_ toxicity. Astrocyte viability was measured by FDA in the presence of 200µM H _2_O _2_ (2 way ANOVA, TXNIP1 and H _2_O _2_ interaction: F=2.94; TXNIP1 shRNA, F=2.94; H _2_O _2_, p<0.001, F=35.5; post-hoc: **p<0.01 vs control). Right panel: However, neuronal viability is not increased by reduced TXNIP1 in astrocytes as neurons die in the same proportion after H _2_O _2_ challenge. Viability of neurons was determined after co-culture for three days with astrocytes transfected with TXNIP1 shRNA (2 way ANOVA, TXNIP1 and H _2_O _2_ interaction: F=0.93; TXNIP1 shRNA, F=0.0097; H _2_O _2_; p<0.05, F=10.95). **C**) In neurons, only H _2_O _2_ decreases TXNIP1 levels, whereas IGF-I does not (***p<0.001 vs control). **D**) Reduction of TXNIP1 by IGF-I and H _2_O _2_ in astrocytes depends on Ca ^2+^ as in the presence of the calcium chelator BAPTA-AM, the decrease is abrogated. (F=7.226; *p<0.05 and ***p<0.001 vs. control). C=control, I=IGF-I, H=H _2_O _2_, H+I=H _2_O _2_ + IGF-I.

We then determined possible mediators of the anti-oxidative actions of IGF-I on astrocytes. We examined whether modulation of SODs could be involved because these anti-oxidant enzymes constitute an important detoxifying mechanism in cases of excess ROS. We found that cytosolic Cu/ZnSOD was increased by IGF-I, H _2_O _2_, or both ( Figure 5A), while mitochondrial MnSOD was increased only by H _2_O _2_ ( Figure 5B). Thus, increases in SOD levels form part of the astrocyte response to H _2_O _2,_ and IGF-I does not appear to interfere with these enzymes. Because FOXO participates in cellular responses to ROS, we looked for signals downstream of FOXO inactivation by IGF-I such as thioredoxin inhibitor 1 (TXNIP1), a pro-apoptotic protein dependant on FOXO activity and related to anti-oxidant responses ^20^. We first confirmed that in astrocytes TXNIP1 is also controlled by FOXO; i.e.: in astrocytes expressing dominant negative Foxo, TXNIP1 levels were 89% reduced as compared to mock-transfected astrocytes. Accordingly, IGF-I, which inhibits FOXO, also reduced TXNIP1 levels ( Figure 6A). Strikingly, H _2_O _2_, which stimulates FOXO activity in astrocytes ( Figure 3D), also inhibited TXNIP1 ( Figure 6A), suggesting alternative routes of TXNIP1 regulation in the presence of H _2_O _2_. When IGF-I and H _2_O _2_ were simultaneously added to astrocytes, TXNIP1 levels were markedly decreased (p<0.05 vs. IGF-I or H _2_O _2_ alone, Figure 6A). To determine the impact of downregulation of TXNIP1 on astrocyte survival we inhibited its expression with shRNA (blot in Figure 6B left panel) and found that astrocytes became resistant to H _2_O _2_ when TXNIP1 levels were low ( Figure 6B). Overexpression of TXNIP1 did not alter the response of astrocytes to H _2_O _2_ whereas co-culture of neurons with astrocytes depleted of TXNIP1 did not result in enhanced neuronal survival ( Figure 6B right panel), indicating that this route is involved in the response of astrocytes to oxidative stress but not in neuroprotection. Interestingly, in neurons, TXNIP1 was downregulated only in the presence of H _2_O _2_, but not after IGF-I treatment ( Figure 6C). Thus, IGF-I down-regulates TXNIP1 only in astrocytes, not in neurons.

We then analyzed possible pathways involved in the inhibitory effect of H _2_O _2_ and IGF-I on TXNIP1. Using kinase inhibitors we ruled out the idea that the main kinases downstream of the IGF-I receptor or H _2_O _2_ were involved. In fact, inhibition of most of these kinases resulted in altered basal levels of TXNIP1 (not visualized), suggesting that basal levels of this protein are tightly regulated in astrocytes. Other inhibitory drugs of different pathways where IGF-I participates (PKC, PKA, CnA, PDK-1, NFκB, among others) gave similar negative results. However, inhibition of Ca ^++^ flux with 5 µM BAPTA abrogated TXNIP1 decreases in response to either H _2_O _2_ or IGF-I while only slightly, but not significantly affecting basal levels ( Figure 6D).

### IGF-I cooperates with SCF produced by astrocytes to protect neurons against oxidative stress

**Figure 7.**
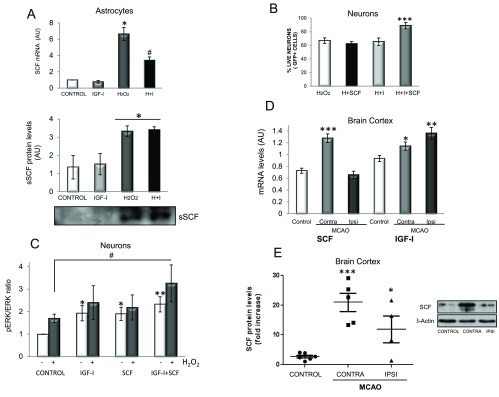
IGF-I cooperates with SCF to promote neuronal survival. **A1**) Upper panel: H _2_O _2_ stimulates SCF mRNA levels in astrocytes after 16 h of exposure whereas IGF-I partially counteracts this increase (F=38.67; *p<0.05 vs. control and IGF-I, #p<0.05 vs. H _2_O _2_). **A2**) Lower panel: H _2_O _2_ stimulates SCF secretion. SCF levels in supernatants from astrocyte cultures treated or not with H _2_O _2_ and/or IGF-I for 24 h. A representative western blot is shown (*p<0.05 vs control). **B**) SCF and IGF-I cooperate to protect neurons from oxidative stress. Neurons were pre-treated with SCF, IGF-I or both 48 h before adding H _2_O _2_ (50 µM) and viability was assessed after overnight treatment (F=12.09, ***p<0.0001 vs H _2_O _2_), H: H _2_O _2_; I: IGF-I. **C**) When H _2_O _2_ is present, Erk phosphorylation is significantly increased only when both SCF and IGF-I are added to the cultures but not with either alone. Neurons were treated with 100 nM IGF-I, 20 ng/ml SCF and 50 µM H _2_O _2_ for 5 minutes and pErk levels were measured by western blot and normalized for total Erk. (*p<0.05 and **p<0.01 vs. control without H _2_O _2_ and #p<0.05 vs. H _2_O _2_). **D**) SCF and IGF-I mRNA levels increased 16 hours after middle cerebral artery occlusion (MCAO) in the contralateral side (CONTRA) in the case of SCF (F=31.53; ***p<0.001 vs. intact control mice) and in both sides in the case of IGF-I (F=7.853; *p<0.05 and **p<0.01 vs. control). **E**) SCF protein levels increase after MCAO in both sides of the cortex (F=12.38; *p<0.05 and ***p<0.001 vs. control). A representative blot is shown. Six, five and four animals were used per group, respectively. Levels of actin in each sample were measured to normalize for total protein levels.

We next analyzed possible neuroprotective effects of IGF-I through astrocytes. Using a commercial gene array for growth factors we screened growth factor production by IGF-I-treated astrocytes in response to H _2_O _2_. Among the several growth factors that increased, stem cell factor (SCF) showed the highest elevation ( Table 3). We confirmed by qPCR that SCF mRNA was increased after H _2_O _2_ whereas IGF-I decreased it ( Figure 7A upper panel). Accordingly, levels of soluble SCF (sSCF) in culture medium from astrocytes treated with H _2_O _2_ were also increased ( Figure 7A, lower panel). As SCF has been shown to be neuroprotective ^21^, we determined whether it protects neurons against H _2_O _2_ and found that while SCF alone did not exert any protection, co-treatment with IGF-I resulted in significantly greater neuronal survival (p<0.05; Figure 7B). We then examined pathways underlying this cooperative action of IGF-I and SCF. Under basal conditions, the activity of extracellular signal-regulated kinase (Erk; measured as pErk/Erk ratio), a canonical kinase in IGF-I signalling, was increased by IGF-I as expected, and to a lesser extent also by SCF ( Figure 7C). Basal Erk activity was also increased by H _2_O _2_. However, Erk was no longer activated by IGF-I or SCF in the presence of H _2_O _2_. Only when both were added together to H _2_O _2_-challenged cultures Erk activity was increased ( Figure 7C). No interactions were found with Akt, the other canonical kinase pathway activated by IGF-I.

**Table 3.** Growth factors array.

Upregulated genes		Downregulated genes	
Gene symbol	Fold regulation	Gene symbol	Fold regulation
Bmp4	34.1544	Amh	**-43.2611**
Bmp8a	19.7667	Bdnf	**-5.4642**
IGF-I	**185.7219**	Bmp1	-51.304
IL7	**54.0417**	Bmp2	-8.3513
Inhbb	**595.9304**	Bmp3	**-311.6969**
SCF	**3072.0799**	Bmp6	-30.211
VEGFb	22.0239	Bmp7	**-40.3361**
		Clcf1	-7.5162
		Csf1	-6.4666
		Csf3	**-102.9643**
		Cxcl1	-35.4079
		Cxcl12	**-49.3166**
		Egf	-24.916
		Ereg	-10.8078
		Fgf1	-11.353
		Fgf10	-15.6273
		Fgf14	-12.7639
		Fgf18	-21.0245
		Fgf2	-9.6934
		Fgf22	-13.5011

A battery of growth factors was screened with an RT ^2^ Profiler ^TM^ PCR Array. In brief, astrocytes were treated or not with IGF-I+H _2_O _2_ for 16 h and total RNA was isolated. After performing the RT-PCR, total cDNA was assayed for PCR Array. PCR data was analyzed with the RT ^2^ Profiler PCR Array Data Analysis version 3.5 software provided by the manufacturer. Significantly up- or downregulated genes are shown.

To determine the *in vivo* relevance of these observations we submitted mice to brain ischemia as this brain insult is associated to oxidative stress ^22^, and both IGF-I ^9^ and SCF ^23^ have been shown to be neuroprotective after ischemia. We found that IGF-I mRNA is increased after middle cerebral artery occlusion (MCAO) both in the ipsilateral and contralateral cortex, while only the contralateral side showed increased SCF mRNA levels compared to intact mice ( Figure 7D). However, levels of SCF protein were elevated after MCAO in both the damaged and contralateral sides compared to normal mice ( Figure 7E). This suggests that after brain ischemia the contralateral cortex produces higher amounts of SCF that eventually reach the ischemic side. Under this condition IGF-I may interact with SCF to promote neuronal survival in the ipsilateral cortex.

Update: Data on the responses of neurons and astrocytes to oxidative injury in the presence of insulin-like growth factor I Fig1a) Neuron viability measured as percentage of GFP positive cells cultured ± astrocytes after treatment with H2O2. N: neurons, NA: neurons + astrocytes. Names for each file refer to the related figures in the associated research article.
Fig1b) IGF-I measured in the supernatant of neuron or astrocyte cultures treated ± with H2O2.
Fig1c) UPDATED with additional data: pAKT levels measured in astrocytes transfected with an IGF-IR dominant negative construct (IGF-IR DN) after treatment with IGF-I.
Fig1d) Neuron viability measured as percentage of GFP positive cells cultured with astrocytes transfected with IGF-IR DN.
Fig2a) pAKT levels in astrocytes treated ± with PPP (picropodophyllin) after treatment with IGF-I.
Fig2b) Neuron viability measured as percentage of GFP positive cells treated ± with PPP and H2O2.
Fig2c) Neuron viability expressed as percentage of GFP positive cells cultured with astrocytes and treated ± with PPP and H2O2.
Fig2d) Neuron viability measured as percentage of GFP positive cells cultured with astrocytes (both cells types from forebrain) and treated ± with PPP and H2O2.
Fig2e) Neuron viability measured as percentage of GFP positive cells cultured with astrocytes and treated with IGF-I, H2O2 or both.
Fig3a) Neuron death measured as percentage of PI positive cells treated ± with IGF-I and H2O2.
Fig3b) Astrocyte survival expressed as percentage of PI negative cells treated ± with IGF-I and H2O2.
Fig3c) FOXO activity from astrocytes treated with IGF-I, H2O2 or both.
Fig3d) Cell survival expressed as percentage of GFP positive cells for astrocytes transfected with wt or AKT-insensitive mutant FOXO, treated ± with IGF-Iand H2O2.
Fig3e) pAKT levels form astrocytes treated with IGF-I I ± H2O2.
Fig4a) Mitochondrial O2- measured in astrocytes treated ± with IGF-I and H2O2.
Fig4b) Total ROS (reactive oxygen species) measured in astrocytes treated ± with IGF-I and H2O2.
Fig5a) Cu/Zn SOD (superoxide dismutase) levels from astrocytes treated with IGF-I, H2O2 or both.
Fig5b) Mn SOD levels from astrocytes treated with IGF-I, H2O2 or both.
Fig6a) TXNIP levels from astrocytes treated with IGF-I, H2O2 or both.
Fig6b) Cell survival from astrocytes transfected with TXNIP shRNA ± H2O2.
Fig6c) TXNIP levels from neurons treated with IGF-I, H2O2 or both.
Fig6d) TXNIP levels from astrocytes pre-treated with the calcium inhibitor BAPTA in the presence of IGF-I, H2O2 or both.
Fig7a) SCF (stem cell factor) mRNA from astrocytes treated with IGF-I, H2O2 or both.
Fig7b) Neuron viability measured as percentage of GFP positive cells treated with IGF-I, SCF or both in the presence of H2O2.
Fig7c) pERK levels form astrocytes treated with IGF-I, SCF or both ± H2O2.
Fig7d) SCF and IGF-I mRNA from control or MCAO (medial cerebral artery occlusion) animal cortex (contralateral or ispsilateral cortex).
Fig7e) SCF protein levels from control or MCAO animal cortex (contralateral or ispsilateral cortex).
Luminol) Reactive oxygen species (ROS) measurements using luminol (which detects superoxide anions)

## Discussion

The present results indicate that IGF-I exerts a protective action on astrocytes contributing to the resilience of these glial cells against oxidative stress. IGF-I also cooperates with astrocytes to protect neurons. These observations highlight the importance of cell-specific and cell-cooperative aspects of IGF-I protection against oxidative challenge. Thus, a better understanding of the trophic role of IGF-I in the brain requires taking into account its effects on astrocytes (and other brain cells) and the functional links of these cells with neurons. While these observations do not help settle the role of oxidative stress in brain aging they put forward an important aspect of possible mechanisms involved in aging; regulatory signals such as IGF-I may not modulate the response of the different cells and even tissues to oxidative stress in the same way.

**Figure 8.**
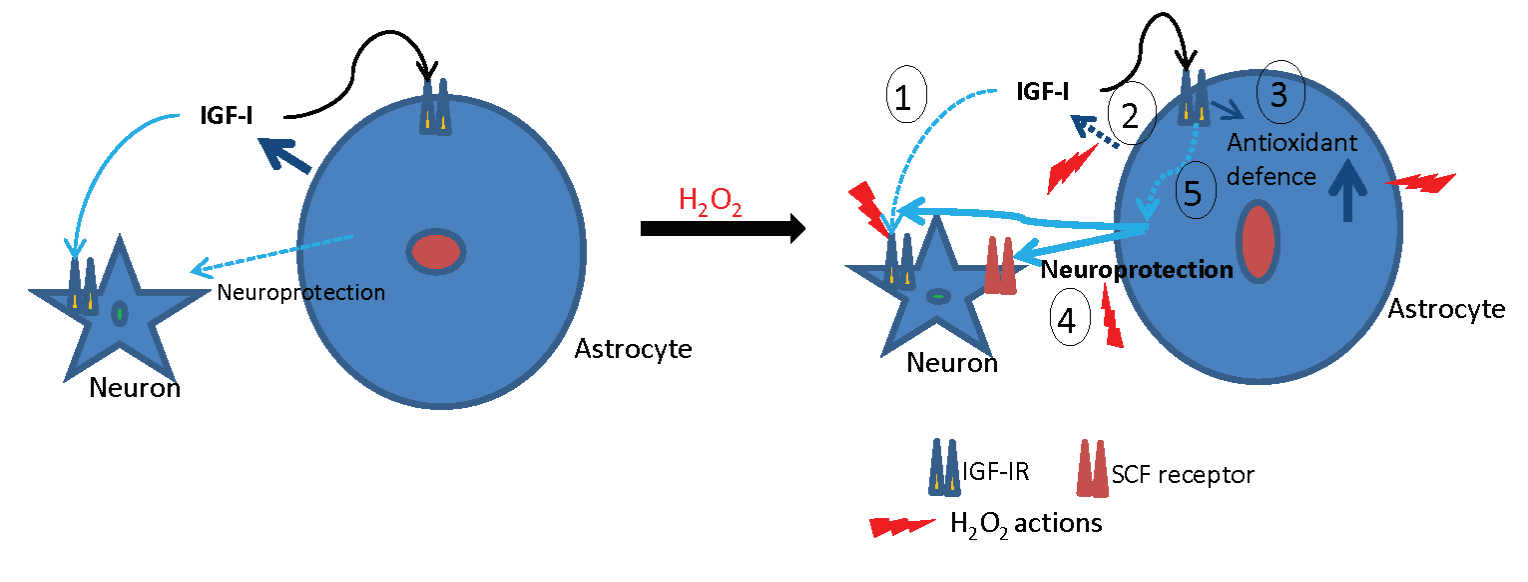
Schematic representation of IGF-I neuroprotection through astrocytes. Left: under basal conditions IGF-I exerts potent neuroprotective actions directly onto neurons, as extensively documented previously ^8^ (also shown in Figure 3A), and probably also through astrocytes. In the presence of H _2_O _2_ (right side) the actions of IGF-I on neurons and astrocytes can be summarized in 5 points: 1) IGF-I loses its ability to directly protect neurons, 2) IGF-I secretion by astrocytes is diminished, 3) IGF-I reinforces astrocyte defences against oxidative stress by down-regulating pro-oxidant mechanisms such as TXNIP1. 4) IGF-I cooperates with SCF secreted by astrocytes to promote neuronal survival. 5) However, the precise mechanism(s) downstream of astrocyte IGF-I receptors underlying enhanced astrocyte neuroprotection remains to be determined. Cytotoxic effects are depicted in red while cytoprotective actions are indicated in blue trace.

The protection provided to astrocytes by IGF-I against oxidative stress may contribute to the greater resilience of these cells to oxidative challenge. In addition, astrocytes are coupled to neurons in the response to oxidative stress and provide them with ample detoxification support ^11^. Among different anti-oxidant defences provided by astrocytes to neurons, we now find that IGF-I, which cannot protect isolated neurons against excess ROS ^12^ cooperates with SCF secreted by astrocytes to support neurons ( Figure 8). While in response to oxidative stress the production of IGF-I by cultured astrocytes and neurons is decreased, after brain ischemia IGF-I levels are actually higher due to increased synthesis and accumulation in microglia, vessels and astrocytes ^24^. Therefore, *in vivo*, astrocytes and neurons will receive IGF-I input from various local sources, suggesting that the response of increased IGF-I after brain ischemia reflects an endogenous neuroprotective mechanism against oxidative injury. This conclusion apparently contradicts other evidence that IIS activity is pro-oxidant. Thus genetic ablation of ISS components in the nematode *Caenorhabditis elegans* ^25^ or in higher organisms such as the fruit fly ^26^ or mice ^27^, increases organism resistance to oxidative stress. For example, mice with reduced IGF-I activity (hemizygous for the IGF-I receptor) have lower levels of ROS in the brain ^28, 29^. However, these mice developed greater cell damage after oxidative injury ^29^. Conceivably, the effects of modulating IGF-I signalling prior to ROS insult (as when using genetic models) may not be the same compared to after insult. For example IGF-I protects nerve cells and/or the brain against diverse types of ROS-related insults ^30– 34^. In this regard, we recently reported that in a cellular model of Friedreich's ataxia (which elicits oxidative damage) neurons responded to IGF-I only when they became frataxin deficient, but not under normal conditions ^15^. Collectively these observations emphasize the importance not only of cell type but also of context dependency of IGF-I neuroprotection in relation to oxidative stress.

A role for oxidative stress in many neurodegenerative diseases is gaining increasing acceptance ^35^. Aberrant production of ROS in the central nervous system is linked to neurodegenerative diseases such as Alzheimer’s dementia, Parkinson’s disease or stroke, all of them associated to aging ^36^. However, as already commented, the role of oxidative stress in brain aging is still unclear. An attempt to explain these apparently opposing observations is that moderate ROS levels may activate survival pathways ^37^. The present findings agree with this proposal. Thus, doses of H _2_O _2_ up to 100 µM do not elicit astrocyte death probably because IGF-I helps maintain their anti-oxidant capacity. In this regard our results show that astrocytes in response to IGF-I and/or H _2_O _2_ activate antioxidant signalling including upregulation of Cu/ZnSOD and MnSOD coupled to downregulation of pro-oxidant proteins such as Txnip1. Txnip1 inhibits thioredoxin (Trx), a protein that reduces protein disulfides as well as H _2_O _2_. The Txnip-Trx axis plays an important role in different brain diseases in which oxidative stress is implicated ^38^.

There is ample evidence that different trophic factors, including SCF ^39^, contribute to reduce cell damage due to oxidative stress after brain stroke ^40^. We have found that *in vitro* IGF-I and SCF exert a cooperative neuroprotective effect against oxidative stress, suggesting that they may exert a similar beneficial role *in vivo* as after brain stroke both factors are upregulated in the lesioned area. Indeed, a cooperative neuroprotective effect of SCF with insulin has been reported ^41^. The intracellular mechanisms mediating cooperation between these two factors involve Erk, a kinase activated by IGF-I.

In summary, cell specific and cooperative actions of IGF-I in brain responses to oxidative challenge underscores the need to design therapeutic strategies that take into account all aspects of biological organization, leading, for example, to cell-specific targeting of anti-aging drugs.

## Data availability

figshare: Update 1: Data on the responses of neurons and astrocytes to oxidative injury in the presence of insulin-like growth factor I, http://dx.doi.org/10.6084/m9.figshare.991456 ^70^
