# Supplementary material for: Astrocytes require insulin-like growth factor I to protect neurons against oxidative injury
Source: F1000Res. 2014 Apr 22;3:28. Originally published 2014 Jan 28. [Version 2] doi: 10.12688/f1000research.3-28.v2 (PMC3954172; doi:10.12688/f1000research.3-28.v2)
Supplement: Peer review history

# Peer review: Referee response for version 2

**DOI:** 10.5256/f1000research.4206.r4528

**Published:** 2014-05-01

**Authors:** Marta Margeta

**Affiliations:** 1 Department of Pathology, University of California San Francisco, San Francisco, CA, USA

In the revised article, the authors have addressed most of my concerns. There still remain a few relatively minor issues, mostly involving data presentation / manuscript readability rather than scientific validity.

1. As in the prior version of the manuscript, the authors claim in Fig. 8 (model figure) that IGF-1 has a neuroprotective effect under basal conditions (i.e. in the absence of H _2_O _2_) and now explicitly cite Fig. 3A in support of this claim. However, as previously, Fig. 3A includes no information on the statistical significance of IGF-1 effect on the neuronal death in the absence of H _2_O _2 _(by eye, the IGF-1 effect seems very modest). In the legend for Fig. 3A, the authors state that "#" means " *p<0.05 vs control*" but symbol "#" is not used in the figure - perhaps it has been accidentally omitted?
2. I appreciate that, as I suggested, the authors repeated statistical analyses for many experiments and are now using two-way rather than one-way ANOVA where appropriate. However, the results of this analysis are buried in the figure legends and are described in a fairly non-transparent, difficult to understand way; the figures themselves are largely unmodified. To make the paper more accessible to future readers, the graphs for experiments analyzed by 2-way ANOVA should show statistical significance for all post-hoc comparisons that were performed (including the effects that were not significant, to make this clear); for a 2x2 experimental condition grid this means all 4 post-hoc comparisons should be shown in terms of statistical significance. As an example, for the experiment in Fig. 2B, the authors should show statistical significance for (1) H _2_O _2_ vs vehicle in control; (2) H _2_O _2_ vs. vehicle following PPP pre-treatment; (3) control vehicle vs. PPP vehicle; and (4) control H _2_O _2_ vs. PPP H _2_O _2_. Analogous pairwise comparisons should be shown for all experiments using 2-way ANOVA.

I have read this submission. I believe that I have an appropriate level of expertise to confirm that it is of an acceptable scientific standard.

# Response

**Published:** 2014-05-09

**Authors:** Ignacio Torres Aleman

**Affiliations:** Cajal Institute, Spain

In response to the minor points raised by the reviewer:

1. The symbol # in Figure 3A is missing due to a typographical error.
2. We now incorporate the requested table for the experiments analyzed by 2-way ANOVA including non-significant values.

# Peer review: Referee response for version 1

**DOI:** 10.5256/f1000research.3363.r3862

**Published:** 2014-03-06

**Authors:** Vince C Russo

**Affiliations:** 1 Centre for Hormone Research, Murdoch Children’s Research Institute, Parkville, VIC, Australia

This manuscript by Laura Genis and co-workers is very interesting and the data presented are of significant scientific value. These studies provide further understanding of the cellular and molecular mechanisms involved in neuronal damage and rescue following oxidative stress. Of particular interest are the protective effects of IGF-I on neuronal cells mediated via the astrocytes. To these, the synergistic/additive effects of SCF on IGF-I are novel and interesting.

I have only few minor suggestions - mainly to improve graphic illustrations:

1. In figure 1B, IGF-I ELISA, the conditioned media from astrocytes and neuronal cells is analysed for IGF-I levels but the values are express in ng/ug of protein, why?
2. In Figure 3E I asume that the graph shows the % phosphorylation for the IGF-I treatment? This should be properly label.
3. In Figure 4 a statistical significance is shown for the 'untreated', but it should shown for the IGF-I which actually prevents MitoO2. I have a similar comment for the graph below in figure 4B.

I have read this submission. I believe that I have an appropriate level of expertise to confirm that it is of an acceptable scientific standard.

# Peer review: Referee response for version 1

**DOI:** 10.5256/f1000research.3363.r3443

**Published:** 2014-02-07

**Authors:** Carlos Matute

**Affiliations:** 1 Department of Neuroscience, País Vasco University, Leioa, Spain

This is an excellent paper with data relevant to CNS protection against oxidative stress.

I have read this submission. I believe that I have an appropriate level of expertise to confirm that it is of an acceptable scientific standard.

# Peer review: Referee response for version 1

**DOI:** 10.5256/f1000research.3363.r3440

**Published:** 2014-02-06

**Authors:** Marta Margeta

**Affiliations:** 1 Department of Pathology, University of California San Francisco, San Francisco, CA, USA

In this paper, the authors attempt to elucidate the role of IGF-I in the astrocyte-mediated protection of neurons against oxidative stress. While this is an important topic and the authors present a lot of interesting data, the paper is unfocused and the results do not fully support the conclusions. As such, I feel that the paper cannot be approved for indexing until substantively revised.

**Major comments:**

1. The paper presents three essentially independent sets of data and then tries to connect them into a single coherent story, without direct experimental evidence that it is appropriate to do so. Specifically, Figs. 1 and 2 present evidence that IGF-I plays an important role in the astrocyte-mediated neuroprotection, both at baseline conditions and under oxidative stress; this is the most interesting part of the paper (and the part that is most relevant to the paper’s current title). Figs. 3-6 show data that elucidate some aspects of IGF-I effect on astrocytes, but do not establish the importance of these effects/mechanisms for IGF-I- and astrocyte-mediated neuroprotection. (Notably, the authors have actually established that one of these mechanisms, IGF-I-induced decrease in the expression of astrocytic TXNIP1, does not play a role in the astrocyte-mediated neuroprotection. Surprisingly, these key results are not shown despite the fact that an entire figure [Fig. 6] is devoted to the IGF-I modulation of TXNIP1).
  Finally, Fig. 7 shows that IGF-I and SCF applied together (but not separately) have a neuroprotective effect in the absence of astrocytes and that their expression is increased in an *in vivo* stroke model. However, the authors again fail to show that these observations are in any way relevant for the astrocyte-mediated neuroprotection shown in Figs. 1 and 2. To connect these currently unconnected experimental threads, the authors need to use their neuron-astrocyte co-culture system to establish the link between astrocyte-mediated neuroprotection and (1) IGF-I-mediated increase in the expression of astrocyte antioxidant enzymes, (2) IGF-I-mediated decrease in the astrocyte ROS levels, and (3) astrocyte secretion of SCF; the experiments should be performed both at baseline and under oxidative stress conditions. Alternatively, the paper needs to be re-written in a way that makes it very clear (1) that the data presented in the paper represent a series of independent observations that do not add up to a coherent whole and (2) that the mechanism mediating IGF-I-induced neuroprotection in the mixed neuron-astrocyte environment currently remains unexplained. If choosing this route, the authors should also change the title of the paper to something more neutral and descriptive.
2. The authors do not show some important experimental data, ostensibly “for clarity”; these data need to be included in the revised paper. Specifically, as already stated in point #1, the authors mention (but do not show) that depleting astrocytes of TXNIP1 does not result in increased neuronal survival (page 16). This finding, if properly established, indicates that the IGF-I-mediated decrease in TXNIP1 and the IGF-I/astrocyte-mediated neuroprotection are two entirely unrelated phenomena. Given the paper’s overall title and conclusions, this is a key experiment that needs to be shown. Similarly, it is important to show the results of IFG-IR DN experiment performed under oxidative stress conditions (page 7).
3. In many experiments, the authors do not use appropriate statistical analyses. Specifically, given the experimental design, two-way (rather than one-way) ANOVA should be used to analyze data shown in Figs. 1B-C, 1D (after missing data are included), 2A-E, 3A-D, 4A-B, and 6B.
4. Fig. 8 (the model) does not accurately represent the experimental results. For example, the authors state in the Fig. 8 legend that “ *under basal conditions IGF-I exerts potent neuroprotective actions directly onto neurons*” when in fact, IGF-I has no clear neuroprotective effect when applied to neurons cultured alone (Fig. 3A, 0 µM H _2_O _2_ condition) – the IGF-I-induced decrease in neuronal cell death is small [~5% based on the graph] and does not appear statistically significant, although the authors do not comment on this one way or the other. Similarly, the figure legend mentions that IGF-1 down-regulates astrocytic TXNIP1 – a finding that is accurate but not relevant for the astrocyte-mediated neuroprotection illustrated by the figure. Thus, Fig. 8 should either be altered to more meaningfully represent the paper’s findings or, if the authors decide to re-write the paper in a more descriptive fashion, could be eliminated altogether.

**Minor comments:**

1. Why are neurons cultured under depolarizing conditions (25 mM KCl)?
2. The co-culture experimental set-up should be described under a separate heading, not buried under “ **Cell assays**”.
3. For similar experiments, the authors should use a similar type of plot to make the paper more readable. For example, experiments in Figs. 3A and 3B have a very similar overall design – why are the results shown very differently?
4. Neuronal viability is established by counting “ *all GFP-positive cells*”; the authors therefore need to show that their neuronal cultures are at least 95% and preferably 99% pure (i.e., do not contain a significant population of GFP-positive glial cells).
5. Representative flow cytometry plots should be included in Fig. 4A.
6. To enhance readability, all figure panels should be labelled “astrocytes”, “neurons”, or “neurons + astrocytes”, as appropriate. (The information is currently largely buried in the figure legends.)
7. In Fig. 5, authors should specify whether they are measuring mRNA or protein level (I assume the latter, but it’s difficult to be sure).

I have read this submission. I believe that I have an appropriate level of expertise to confirm that it is of an acceptable scientific standard, however I have significant reservations, as outlined above.
